# Co-circulation of monkeypox virus subclades Ia and Ib in Kinshasa Province, Democratic Republic of the Congo, July to August 2024

**DOI:** 10.2807/1560-7917.ES.2024.29.38.2400592

**Published:** 2024-09-19

**Authors:** Tony Wawina-Bokalanga, Prince Akil-Bandali, Eddy Kinganda-Lusamaki, Emmanuel Lokilo, Daan Jansen, Adrienne Amuri-Aziza, Jean-Claude Makangara-Cigolo, Elisabeth Pukuta-Simbu, Rilia Ola-Mpumbe, Mamito Muyembe, Cris Kacita, Princesse Paku-Tshambu, Pedro HLF Dantas, Olivier Tshiani-Mbaya, Gradi Luakanda, Antoine Nkuba-Ndaye, Meris Matondo, Emmanuel Hasivirwe Vakaniaki, Sofonias Tessema, Nicaise Ndembi, Áine O’Toole, Tessa De Block, Christian Ngandu, Nicole A Hoff, Nicola Low, Lorenzo Subissi, Sydney Merritt, Jean-Jacques Muyembe-Tamfum, Laurens Liesenborghs, Martine Peeters, Eric Delaporte, Jason Kindrachuk, Anne W Rimoin, Steve Ahuka-Mundeke, Andrew Rambaut, Dieudonné Mwamba, Koen Vercauteren, Placide Mbala-Kingebeni

**Affiliations:** 1 Institut National de Recherche Biomédicale (INRB), Kinshasa, Democratic Republic of the Congo; 2 Service de Microbiologie, Département de Biologie Médicale, Cliniques Universitaires de Kinshasa, Université de Kinshasa, Kinshasa, Democratic Republic of the Congo; 3 Department of Clinical Sciences, Institute of Tropical Medicine, Antwerp, Belgium; 4 TransVIHMI, Université de Montpellier, INSERM, IRD, Montpellier, France; 5 Graduate School of Cellular and Biomedical Sciences, University of Bern, Bern, Switzerland; 6 Institut National de Santé Publique (INSP), Kinshasa, Democratic Republic of the Congo; 7 Africa Centres for Disease Control and Prevention, Addis Ababa, Ethiopia; 8 Institute of Ecology and Evolution, University of Edinburgh, Edinburgh, United Kingdom; 9 Department of Epidemiology, Jonathan and Karin Fielding School of Public Health, University of California, Los Angeles, CA, United States; 10 Institute of Social and Preventive Medicine, University of Bern, Bern, Switzerland; 11 World Health Organization, Geneva, Switzerland; 12 Department of Microbiology, Immunology and Transplantation, KU Leuven, Leuven, Belgium; 13 Department of Medical Microbiology & Infectious Diseases, University of Manitoba, Winnipeg, Manitoba, Canada; Department of Internal Medicine, University of Manitoba, Winnipeg, Manitoba, Canada; * These authors contributed equally to this work and share last authorship.

**Keywords:** Mpox, Monkeypox virus (MPXV), Co-circulation, Clade I, Kinshasa, Democratic Republic of the Congo, public health policy, surveillance

## Abstract

Between January and August 2024, mpox cases have been reported in nearly all provinces of the Democratic Republic of the Congo (DRC). Monkeypox virus genome sequences were obtained from 11 mpox cases’ samples, collected in July–August 2024 in several health zones of Kinshasa. Characterisation of the sequences showed subclades Ia and Ib co-circulating in the Limete health zone, while phylogenetic analyses suggested multiple introductions of the two subclades in Kinshasa. This illustrates the growing complexity of Clade I mpox outbreaks in DRC.

Mpox is a zoonotic disease caused by monkeypox virus (MPXV), which is endemic to Western and Central African countries and has the highest prevalence in the Democratic Republic of the Congo (DRC) [1]. There are two clades of MPXV, namely Clade I and Clade II, each respectively divided into two subclades a and b [2-4]. The geographic range of Clade I, and predominantly subclade Ib within, is currently expanding, with cases reported in countries historically non-endemic for mpox, including Burundi, Kenya, Rwanda and Uganda [5]. Travel-associated transcontinental importations have also occurred in Sweden and Thailand [6]. In this context, the situation regarding mpox in Kinshasa, the capital city of the DRC, represents a particular concern due to the city’s (i) large population (> 17 million inhabitants) and population density, (ii) proximity to Brazzaville, the capital of the Republic of the Congo and (iii) international connections possible via air travel.

Here, characterisation of complete MPXV genome sequences derived from mpox cases in Kinshasa reveals co-circulation MPXV strains of subclades Ia and Ib in one health zone of the city. Moreover, phylogenetic analyses suggest multiple introductions of both subclades in the city.

## Mpox case sample collection, laboratory processing and bioinformatic analyses

Kinshasa, which is both a city and a province, is divided into 35 health zones in terms of healthcare services. All samples collected from suspected cases of mpox across the province are sent to the Institut National de Recherche Biomédicale (INRB), Kinshasa. 

As part of routine country-wide mpox surveillance, the INRB laboratory received 12 samples from 11 suspected mpox cases in Kinshasa, reported between July and mid-August 2024. The samples originated respectively from vesicle (n = 7) and crust (n = 4) swabs, along with one swab of conjunctival secretions. They had been collected by local surveillance teams, who provided data from each suspected case using the national investigation form. This form includes information on demographic characteristics (age, sex, residence, including health zone and province, profession and nationality), time of onset of clinical symptoms, type of sample and sampling date of cases.

Viral DNA was extracted from 140 µL of inactivated swab material resuspended in 1× phosphate buffered saline (PBS) solution using the QIAamp DNA Mini Kit (Qiagen, Hilden, Germany), following the manufacturer’s instructions. Real-time PCR assays were performed using both Orthopoxvirus- and MPXV-generic primers/probes for mpox diagnosis [7,8]. The 12 samples from the 11 cases were subjected to sequencing; two samples, identified as 24MPX-1521GG and 24MPX-1521V, were collected from the same patient. To sequence the full-length MPXV genome, we used either the Comprehensive Viral Research Panel (Twist Biosciences) or the Clade I-optimised tiling sequencing protocol, which is a modified version of the Welkers et al. protocol (https://www.protocols.io/view/monkeypox-virus-whole-genome-sequencing-using-comb-n2bvj6155lk5/v1). Sequencing libraries were loaded onto either the MiSeq or GridION sequencer. FASTQ files from the MiSeq were processed using GeVarLi (https://forge.ird.fr/transvihmi/nfernandez/GeVarLi), CZ ID (https://czid.org/), and iVar pipelines. FASTQ files from the GridION were processed with fastp to trim adapter sequences and filter out low-quality bases [9]. The trimmed reads were aligned to the human genome using Minimap2 [10], and those that aligned were removed. The remaining reads were classified with MetaMaps (https://github.com/DiltheyLab/MetaMaps) against the viral RefSeq database. Reference-based (GenBank ID: NC_003310) consensus genomes were built using iVar (https://github.com/andersen-lab/ivar), and indels were filtered out using Homopolish (https://github.com/ythuang0522/homopolish).

Clade assignment was performed using Nextclade tool (https://clades.nextstrain.org/).

Multiple sequence alignment against the Clade I MPXV reference genome (GenBank ID: NC_003310) was performed using SQUIRREL (https://github.com/aineniamh/squirrel). We Inferred a maximum-likelihood phylogenetic tree using IQ-TREE2 v2.1.4 [11] with the K3Pu + F + I’ substitution model as the best fit. Branch support was estimated by the ultrafast bootstrap approximation with 10,000 replicates [12].

## Description of cases

**Figure 1:**
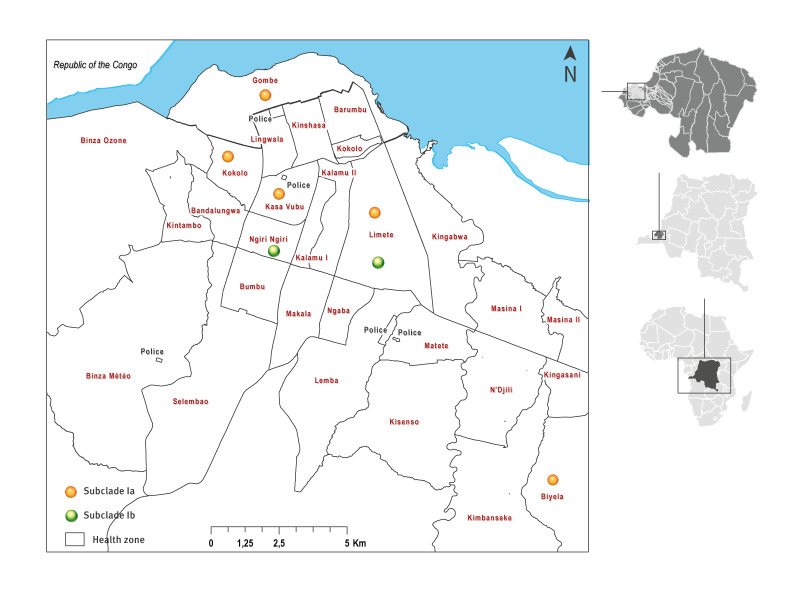
Geographic distribution of mpox confirmed cases, Kinshasa, Democratic Republic of the Congo, July–August 2024 (n = 11 cases)

All 11 suspected mpox cases were PCR confirmed with quantification cycle (Cq) values ranging from 16.81 to 30.59 for the PCR with the MPXV-generic primers. Five cases were from the Limete health zone and two from the Kasa-Vubu health zone (Figure 1). The four remaining cases were respectively from the Gombe, Kokolo, Biyela, and Ngiri-Ngiri health zones. Overall, two cases were ≤ 10 years-old, while the remainder were evenly distributed in 11–21, 22–32 and 33–43 year-age groups, which comprised three cases each. In total, seven cases were of male and four of female sex.

Names of the health zones figure in red font. Both subclades Ia and Ib are co-circulating in the health zone of Limete. A mono-circulation pattern of subclade Ia is observed in the following health zones: Gombe, Kokolo, Kasa-Vubu, Biyela, while a mono-circulation pattern of subclade Ib is observed in Ngiri-Ngiri. Blue colour indicates the Congo River.

MPXV genomes were generated from all 12 samples, collected from the 11 cases, with horizontal genome coverage ranging from 68.5% to 98.8%. Seven MPXV genomes were characterised as subclade Ia and five as subclade Ib MPXV (Table). MPXV subclade Ib was detected in age groups of 0–10, 11–21, and 22–32 years old. In the health zones of Biyela, Gombe, Kasa-Vubu and Kokolo, only viruses of subclade Ia were detected, while the single sample from Ngiri-Ngiri harboured a subclade Ib virus. In the Limete zone three cases had been infected by subclade Ib viruses and two by subclade Ia viruses (Figure 1).

**Table:** Cq values obtained by a PCR with MPXV-generic primers and genome coverage of MPXV genomes derived from samples of mpox cases, Democratic Republic of the Congo, July–August 2024 (n = 12 samples)^a^

Sample ID	Sample type	Cq value	Consensus genome coverage	Subclade^b^
24MPX-1978V	Vesicle	17.15	98.82%	Ia
24MPX-2056V	Vesicle	17.01	95.78%	Ia
24MPX-1956V	Vesicle	19.46	93.77%	Ib
24MPX-2019V	Vesicle	17.85	90.40%	Ib
24MPX-2027V	Vesicle	21.64	89.10%	Ib
24MPX-2026C	Crust	16.81	88.60%	Ia
24MPX-1521GG^a^	CSS	26.92	88.06%	Ib
24MPX-1521V^a^	Vesicle	17.77	85.83%	Ib
24MPX-2024C	Crust	17.81	84.70%	Ia
24MPX-2091C	Crust	29.47	76.08%	Ia
24MPX-2092C	Crust	30.59	73.30%	Ia
24MPX-2057V	Vesicle	18.58	68.55%	Ia

Cq: quantification cycle; CSS: conjunctival secretion swab; ID: identification; MPXV: monkeypox virus.

^a^ There were 12 samples from a total of 11 mpox cases. Samples identified as 24MPX-1521GG and 24MPX-1521V were collected from the same case.

^b^ Subclades were determined using Nextclade (https://clades.nextstrain.org).

## Phylogenetic analysis of monkeypox virus strains affecting the cases

**Figure 2:**
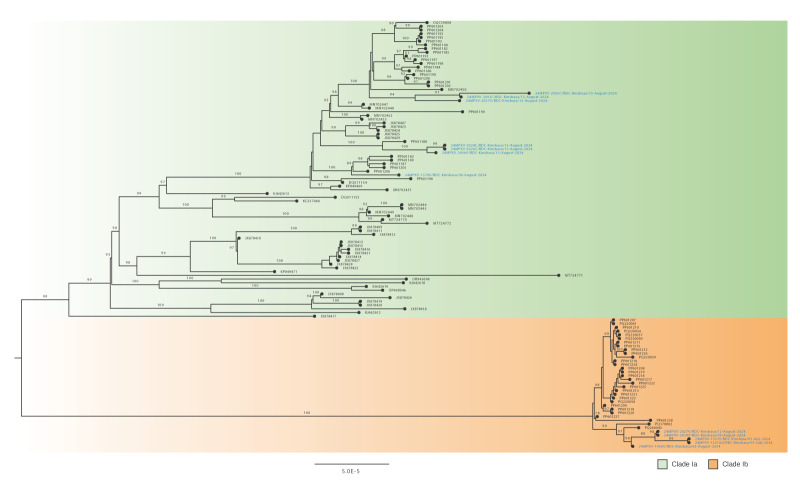
Phylogenetic tree of MPXV sequences (highlighted in blue) from confirmed cases of mpox, Kinshasa, Democratic Republic of the Congo, July–August 2024 (n = 11 cases)

Although contact-tracing information of mpox confirmed cases was incomplete, the constructed phylogenetic tree (Figure 2) suggests multiple independent introductions of both subclades Ia and Ib MPXV, in Kinshasa.

Sequences are divided into two subclades: subclade Ia (green) and subclade Ib (orange). Genomes identified as 24MPX-1521GG and 24MPX-1521V were collected from the same patient.

Concerning the subclade Ia genomes characterised in the current study: 24MPX-2092C and 2091C were both from samples collected in the Kasa-Vubu health zone, and they cluster with 24MPX-2057V, which is from the Biyela health zone; both 24MPX-2024C and 2026C are from the Limete health zone and they cluster with 24MPX-2056V from the Gombe health zone; 24MPX-1578V sequence is from the Kokolo health zone. For the subclade Ib, all genomes characterised in the current study are from the Limete health zone, except for 24MPX-1521V and 24MPX-1521GG which are from the Ngiri-Ngiri health zone.

## Discussion

Since the first human case of mpox was identified in the DRC in 1970 [13], efforts to curb the spread of MPXV have been ongoing. Nevertheless, the number of reported cases has continued to increase over time [14]. According to the DRC Ministry of Public Health, from 1 January to 14 September 2024, there have been 25,757 suspected cases of mpox reported across nearly all 26 provinces, with 806 deaths, resulting in a case fatality (CF) of 3.1%.

In 2022, MPXV caused a large outbreak affecting many countries, leading the World Health Organization (WHO), at the time, to declare this outbreak a public health emergency of international concern (PHEIC) [15]. Prior to this, MPXV was classified into two major clades: Clade I which has historically been reported as associated with more severe disease and a higher overall CF, and Clade II, which is typically associated with milder symptoms [16]. Each of these clades is further subdivided: Clade II into IIa and IIb, with IIb variants associated with the 2022 global mpox outbreak driven by human-to-human transmission [2] and Clade I into Ia and Ib, with Ib variants identified in 2023 [3,4].

While subclade Ia MPXV strains remain most commonly reported in DRC, those of subclade Ib have been receiving increased attention due to their recent spread [5,6]. The subclade Ib has been associated with sustained human-to-human transmission driven by close contact with infected individuals, including through sexual contact [3,4]. Since its emergence in 2023 in South Kivu province, eastern DRC [3,4], mpox cases caused by MPXV of subclade Ib have been reported in North Kivu province (https://virological.org/t/mpox-clade-ib-cases-in-goma/962) and also detected in multiple international locations [5,6], raising global public health concerns. On 13 August 2024 the Africa Centres for Disease Control and Prevention (Africa CDC) declared its first public health emergency of continental security [17], and on 14 August 2024, the WHO declared a PHEIC regarding mpox for the second time since 2022 [18].

In this study, mpox cases were confirmed in six of the 35 health zones of Kinshasa. Whereas we previously described different groups of subclade Ia lineages co-circulating in five health zones of the city, including Gombe, Lemba, Limete, Matete, and Nsele [19], we now demonstrate for the first time, a co-circulation of both subclades Ia and Ib, among five cases detected from the Limete health zone.

While surveillance of mpox cases continues in the different health zones of Kinshasa, we cannot exclude the possibility of undetected mpox cases. A limitation of the study is the lack of comprehensive contact-tracing information for all mpox confirmed cases.

## Conclusion

This report describes co-circulation in Kinshasa of the two distinct MPXV subclades Ia and Ib between July and August 2024, illustrating the complexity of mpox outbreaks in DRC. Ongoing genomic investigations are expected to yield more insights into the circulation of these subclades across different provinces of the country. We, therefore, advocate for enhanced surveillance and further epidemiological investigations within the community to better understand and address the factors contributing to mpox outbreaks.

## †Addendum

The GenBank accession numbers of the genomic sequences obtained in the current study were added to this report on 25 September 2024.
