# Addendum for Euro Surveill. 2024;29(38)

**DOI:** 10.2807/1560-7917.ES.2024.29.21.240906A

**Published:** 2024-09-26

**Authors:** Eurosurveillance editorial team

**Affiliations:** 1 European Centre for Disease Prevention and Control (ECDC), Stockholm, Sweden

In the article ‘Co-circulation of monkeypox virus subclades Ia and Ib in Kinshasa Province, Democratic Republic of the Congo, July to August 2024’ by Wawina-Bokalanga et al. published on 19 September 2024, the GenBank accession numbers of the genomic sequences obtained in the study were added to the Data availability statement on 25 September 2024.

